# Global Identification of Anti-Melanoma Cellular Targets by Photochemically Induced Coupling of *L-Shikonin* Reactions on the Surface of Magnetic Particles

**DOI:** 10.3390/pharmaceutics16121543

**Published:** 2024-12-02

**Authors:** Min Li, Wenying Li, Fang Xu, Yiping Pu, Jianguang Li

**Affiliations:** 1 College of Pharmacy, Xinjiang Medical University, Urumqi 830011, China; limin1120@xjmu.edu.cn (M.L.); wenyingli599@163.com (W.L.); xufang5660@126.com (F.X.); 2 Xinjiang Key Laboratory of Natural Medicines Active Components and Drug Release Technology, Xinjiang Medical University, Urumqi 830011, China; 3 Dingxi Maternal and Child Health Hospital, Dingxi 743099, China; 4 College of Pharmacy, Xinjiang Second Medical College, Karamay 834000, China

**Keywords:** *L-Shikonin*, surface grafting, coupling molecules in medicine, target fishing hook, magnetic nanoparticle, melanoma

## Abstract

**Background:** *L-Shikonin*, an active component of Arnebia euchroma (Royle) Johnst., has remarkable pharmacological effects, particularly in its anti-tumour activity. Nonetheless, the specific targets and mechanisms of action remain to be further explored. **Methods:** A novel Fe_3_O_4_@*L-Shikonin* was designed and synthesized in this study by linking Fe_3_O_4_ and *L-Shikonin* through benzophenone. Fe_3_O_4_@*L-Shikonin* was characterized using several techniques, including scanning electron microscopy (SEM), Fourier-transform infrared spectroscopy (FT-IR), and drug removal methods, to determine the content of *L-Shikonin* on the surface of the magnetic particles. Target hooking technology was utilized to identify the target proteins of the compound in melanoma. The synthesized Fe_3_O_4_@*L-Shikonin* was co-incubated with A375 cell lysate, followed by the target proteins, which were purified by magnetic enrichment using magnetic microspheres and identified by high-resolution mass spectrometry. **Results:** AutoDock Vina software was employed for molecular docking analysis, where it was found that *L-Shikonin* targets RPN1, CPEB4, and HNRNPUL1 proteins. Subsequently, A375 cells were treated with *L-Shikonin* at different concentrations (2.5, 5.0, 10.0 μM) for 48 h, and the expressions of the three proteins were observed. The results showed a significant reduction in the relative expression of CPEB4 in the high-dose group compared to the control group (*p* < 0.01). Moreover, the relative expression of HNRNPUL1 was decreased in the medium- and high-dose groups (*p* < 0.05). **Conclusions:** This study initially revealed from the source that *L-Shikonin* may regulate melanoma-specific markers, melanosomes, tyrosine kinases related to abnormal tyrosine metabolism, and melanoma through multiple targets such as CPEB4 and HNRNPUL1. Proliferation and metastasis work together to exert an anti-melanoma mechanism, which provides a new idea for the follow-up study of the molecular pharmacological mechanism of the complex system of total naphthoquinones in *Arnebia euchroma* (Royle) Johns.

## 1. Introduction

Malignant melanoma (MM) is an epithelial malignant tumour originating from melanocytes in the neural crest and arising from the abnormal proliferation of melanocytes and malignant changes in pigmented nevi [1]. The morbidity and mortality of MM are increasing significantly worldwide, accounting for 1% to 3% of all malignant tumours, but with a growth rate of 6% to 7% year by year [2,3]. MM has a poor clinical prognosis due to its high malignancy and susceptibility to blood and lymphatic metastases, so the search for highly effective and less toxic therapeutic drugs is of great importance for clinical needs.

**Figure 1:**
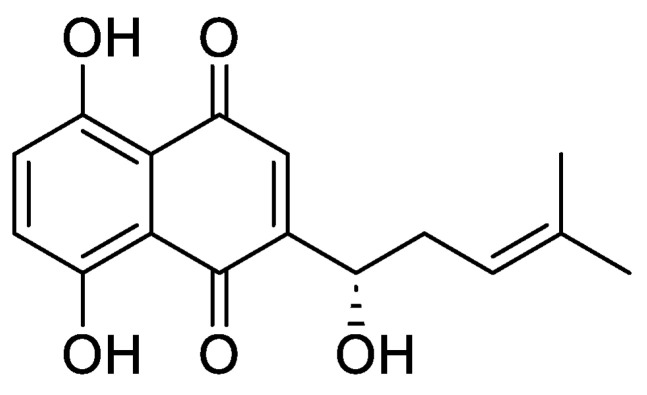
*L-Shikonin* structure.

Arnebia, as a common herb, is widely used in traditional Chinese medicine. According to the regulations of *The Pharmacopoeia of the People’s Republic of China* (2020 edition), Arnebiae Radix is the dried root of the Boraginaceae family plant *Arnebia euchroma* (Royle) Johnst. and *Arnebia guttata* Bunge [4]. Numerous studies have demonstrated the anti-inflammatory, anti-tumour, and antibacterial properties of *A. euchroma* (Royle) Johnst [5,6,7,8,9]. *L-Shikonin*, a natural naphthoquinone compound derived from *Arnebia*, contains a nuclear structure of 5,8 dihydroxy naphthoquinone (Figure 1) [10]. This category of naphthoquinone compounds has demonstrated the ability to impede the growth of various tumour cells and elicit therapeutic outcomes through the induction of tumour cell apoptosis and necrosis [11,12,13,14,15,16,17,18,19]. Furthermore, the inhibitory impact of *L-Shikonin* on melanoma has been validated; however, further investigation is required to elucidate their specific inhibitory mechanism on melanoma [20,21,22,23].

**Figure 2:**
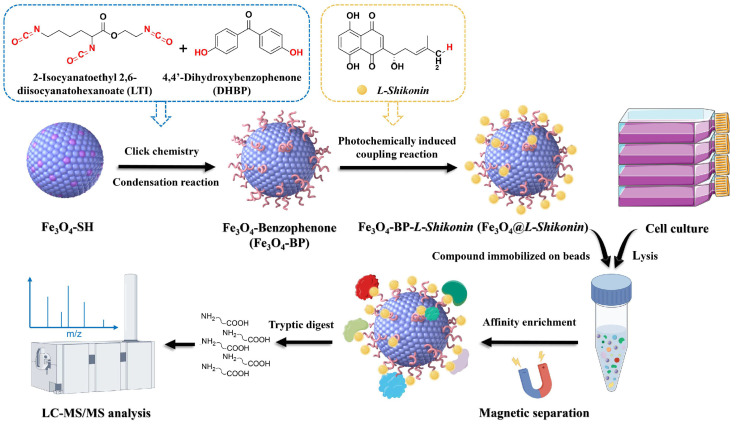
Schematic diagram of the Fe_3_O_4_@*L-Shikonin* structure exhibits the ability to detect cellular targets. On the surface of Fe_3_O_4_-SH, sulfhydryl groups react with the isocyanate groups of 2-isocyanatoethyl 2,6-diisocuanatohexanoate (LTI) via click chemistry. Subsequently, a condensation reaction occurs between the isocyanate groups at the opposite end of LTI and the hydroxyl groups of hydroxyl-containing 4,4′-dihydroxybenzophenone (DHBP), culminating in the creation of Fe_3_O_4_@*L-Shikonin.*

Determining the drug target is one of the most crucial tasks in studying drug mechanisms. This provides an initial biological basis for disease treatment [24]. Molecular target hook fishing technology is crucial for identifying drug targets. It is primarily based on the small molecule affinity chromatography method. This method works on the principle that proteins can specifically bind to drug molecules by chemically attaching them to functional groups on the surface of solid-phase microspheres [25]. Target proteins are captured and enriched on the surface of solid-phase microspheres, making it easier to isolate, purify, and identify them [26]. This method is a widely used drug molecular target identification method, and it is a target identification technology with strong operability. It has effectively promoted the elucidation of the pharmacological mechanism of traditional Chinese medicine and accelerated the modernization process of traditional Chinese medicine [27,28,29,30,31,32,33,34,35]. In this study, Fe_3_O_4_-SH particles were used in the sequential condensation polymerization of 2-isocyanatoethyl 2,6-diisocyanatohexanoate (LTI) and hydroxyl-containing 4,4′-Dihydroxybenzophenone (DHBP) to synthesize a magnetic microsphere functionalized with photosensitive benzophenone groups. Subsequently, *L-Shikonin* was immobilized onto the microspheres’ surface, followed by incubation with melanoma cell lysate for the enrichment and identification of specific target groups using high-resolution mass spectrometry. Through an analysis of the biological functions of individual targets within the target group, this study elucidated the molecular mechanism by which *L-Shikonin* acts against melanoma (Figure 2). The findings offer a novel research perspective on the pharmacological mechanisms underlying *L-Shikonin*’s anti-melanoma effects and hold relevance for understanding the action mechanisms of naphthoquinone compounds in traditional Chinese medicine.

## 2. Materials and Methods

### 2.1. Chemicals and Reagents

The chemicals and reagents used in this study include meso-2,3-dimercaptosuccinic acid (Aladdin Biochemical Technology Co., Ltd., Shanghai, China), 4,4′-dihydroxybenzophenone (Aladdin Biochemical Technology Co., Ltd., Shanghai, China), 1,8-diazabicyclo [5.4.0]undec-7-ene (Aladdin Biochemical Technology Co., Ltd., Shanghai, China), 2-isocyanatoethyl 2,6-diisocuanatohexanoate (Rundong Chemical Co., Ltd., Quzhou, China), sodium hydroxide (Damao Chemical Reagent Factory, Tianjin, China), methanol (Yili Chemical Reagent Co., Ltd., Beijing, China), ethanol (Yili Chemical Reagent Co., Ltd., Beijing, China), acetone (Yili Chemical Reagent Co., Ltd., Beijing, China), dimethyl sulfoxide (Sigma, St. Louis, MO, USA), *L-Shikonin* (purity ≥ 99.0%, Herbpurify Co., Ltd., Chengdu China), DMEM medium (Biological Industries, Kibbutz Beit Haemek, Israel), foetal bovine serum (Biological Industries, Kibbutz Beit Haemek, Israel), penicillin–streptomycin mixture (Biological Industries, Kibbutz Beit Haemek, Israel), 0.25% trypsin (Biological Industries, Kibbutz Beit Haemek, Israel), PBS buffer (Biological Industries, Kibbutz Beit Haemek, Israel), RIPA buffer (Solarbio, Beijing, China), phosphatase inhibitor (Solarbio, Beijing, China), and Fe_3_O_4_-SH particles (Taoyu International Trade Co., Ltd., Shanghai, China). The antibodies used in the study included the primary antibody against RPN1 and HNRNPUL1 (Abcam, Cambridge, MA, USA), the primary antibody against CPEB4 and GAPDH (Bioss Antibodies, Beijing, China), and horseradish peroxidase (HRP)-conjugated secondary antibody (Abcam, Cambridge, MA, USA).

### 2.2. Cell Culture

Human melanoma A375 cells and Human Keratinocytes Cells (Hacat) were kindly provided by the China Type Culture Collection (Beijing, China) and maintained in high-glucose DMEM medium supplemented with 10% foetal bovine serum, 100 U/L penicillin, and 100 μg/L streptomycin at 37 °C in a 5% CO_2_ humidified atmosphere for passaging every 2 days.

### 2.3. Cell Viability Assessment

A375 cells were seeded in a 96-well plate and incubated for 24 h prior to treatment with varying concentrations of *L-Shikonin* (0, 1.0, 2.0, 4.0, 8.0, 16.0, 32.0, and 64.0 μM) for either 24 or 48 h. Cell viability was assessed using the cell counting kit-8 (Bioss Antibodies, Beijing, China) in accordance with the manufacturer’s instructions.

### 2.4. Apoptosis Analysis

A375 cells were cultured in 6-well plates for 24 h, followed by treatment with varying concentrations of *L-Shikonin* (0, 1.0, 5.0, 10.0 μM) for an additional 24 h. Subsequently, the cell apoptosis rate was assessed utilizing an Annexin V/propidium iodide (PI) apoptosis detection kit (Solarbio, Beijing, China) according to the manufacturer’s guidelines.

### 2.5. Cell Cycle Analysis

Following treatment of A375 cells with varying concentrations (2.5, 5.0, and 10.0 μM) of *L-Shikonin* for 24 h, the cells were harvested, rinsed with PBS buffer, and subsequently preserved in ice-cold 70% ethanol overnight at a temperature of 4 °C. Subsequently, the cells underwent a PBS wash and were subjected to staining with RNase and PI for 30 min in the absence of light. The cell cycle distribution was then assessed utilizing flow cytometry (Becton-Dickinson, Franklin Lakes, NJ, USA).

### 2.6. Fe_3_O_4_@L-Shikonin Synthesis

#### 2.6.1. Preparation of DHBP-Bound Fe_3_O_4_ Nanoparticles (NPs)

**Figure 3:**
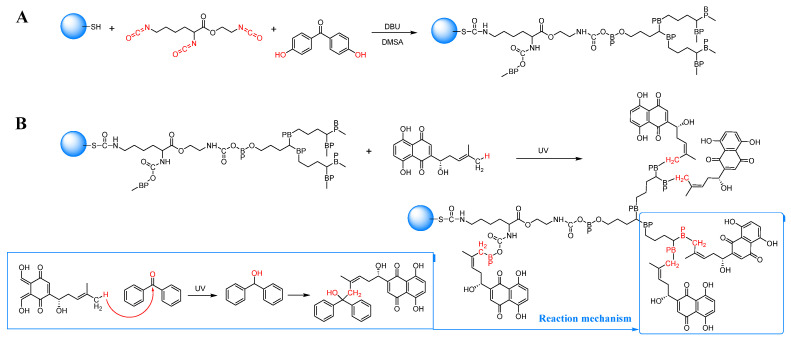
(**A**) Synthesis of Fe_3_O_4_-BP photosensitive magnetic particles through a polymerization reaction. (**B**) *L-Shikonin* binds to the surface of magnetic particles (Fe_3_O_4_-BP) to form drug-conjugated magnetic particles (Fe_3_O_4_-BP-*L-Shikonin*). BP in the chemical structure denotes benzophenone.

The graft polymerization process utilized the surface-SH group of Fe_3_O_4_-SH particles as shown in Figure 3A. A 50 mL dispersion of Fe_3_O_4_-SH particles in acetone was introduced into a 100 mL three-necked flask, followed by stirring at a rate of 200 r/min. Subsequently, monomers LTI and DHBP, along with initiator DBU, were added and allowed to react at room temperature for 0.5 h. The addition of DMSA followed, and the reaction proceeded at room temperature for an additional 2.5 h. Following the reaction, the resulting products were isolated using a magnet and subsequently subjected to six washes with methanol in order to yield magnetic particles (Fe_3_O_4_-BP particles) functionalized with LTI, DHBP, and DMSA polymers, forming a composite known as LTI-DHBP-DMSA. These particles were then dispersed in methanol. 

#### 2.6.2. Chemicals Crosslinked onto DHBP-Bound Fe_3_O_4_ NPs

In a round-bottomed photoreactor, 20 mL of methanolic solution of *L-Shikonin* (0.25 mg/mL) and Fe_3_O_4_-BP particles (15 mg) were added. This mixture was deoxygenated with N_2_ for 30 min and subsequently irradiated under UV light for 1 h using a 375 W mercury lamp with a light intensity of 12.5 W/m^2^ at a wavelength of 254 nm (CME-M500, Zhongke Micro Energy Technology Co., Ltd., Hangzhou, China) to achieve the desired surface conjugation of the drug, as shown in Figure 3B.

### 2.7. Characterization of Magnetic Microspheres

#### 2.7.1. Field-Emission Scanning Electron Microscope (FE-SEM) Analysis

The microstructure of magnetic microspheres was assessed by scanning electron microscopy (JSM-639OLV, JEOL Ltd., Tokyo, Japan). The Fe_3_O_4_-SH magnetic particles and the prepared Fe_3_O_4_@*L-Shikonin* NPs were fixed onto a silicon wafer and imaged under a field-emission scanning electron microscope at an accelerating voltage of 15 kV and a magnification of 25,000×.

#### 2.7.2. Particle Size and Potential Determination

The particle size and zeta potential were further determined using a Nano ZS la-ser particle sizer (Malvern Zetasizer, Malvern, UK).

#### 2.7.3. Ultraviolet–Visible (UV–Vis) Spectrophotometry Analysis

The experiments were conducted using a UV-Vis spectrophotometer (UV-2700, Shimadzu, Kyoto, Japan) in the wavelength range of 200–800 nm. The difference in UV absorption spectra of *L-Shikonin*, Fe_3_O_4_-SH, Fe_3_O_4_-BP, and Fe_3_O_4_-BP-*L-Shikonin* were determined. Additionally, the concentration of magnetic particle-coupled *L-Shikonin* was determined through supernatant analysis and polymer degradation techniques.

#### 2.7.4. Fourier-Transform Infrared (FT-IR) Spectroscopy Analysis

Dried *L-Shikonin*, magnetic Fe_3_O_4_-SH particles, magnetic Fe_3_O_4_-BP particles, and Fe_3_O_4_-BP-*L-Shikonin* (Fe_3_O_4_@*L-Shikonin*) particles were finely ground using an agate mortar and pestle. Subsequently, 2 mg of each sample was mixed with 200 mg of KBr and compressed under pressure using a hydraulic pelletizer to produce thin KBr particles for analysis and then scanned with an infrared spectrometer (Shimadzu, Japan) in the frequency range of 4000–400 cm^−1^, with KBr flakes serving as a background for comparative infrared spectra observation.

### 2.8. Cell Handling

Approximately 10 million A375 cells were harvested and combined with a cell lysis solution containing RIPA, 1% PMSF, and a 2% phosphoprotein inhibitor mixture. The mixture was then subjected to an ice bath for 30 min, followed by centrifugation at 12,000 rpm for 20 min at 4 °C. The resulting supernatant was collected and stored.

### 2.9. Cellular Target Protein Pull-Down

Fe_3_O_4_-SH particles and Fe_3_O_4_-BP-*L-Shikonin* were added to the total protein extract of A375 melanoma cell as control and *L-Shikonin*-bonded groups, respectively. The groups were mixed and incubated at 4 °C for 24 h to effectively capture the target proteins. Afterward, the samples were washed six times with Tris (pH 7.4), magnetic particle washing solution (PBS solution containing 0.1% Triton X-100), cell lysis solution (RIPA:PMSF:phosphatase inhibitor mixture = 100:1:2), and elution buffer [TBST (1X) solution containing 0.01% RIPA] to remove any residual non-specifically bound proteins.

### 2.10. LC-MS/MS Analysis of Target Proteins

The reaction solution was added to the magnetic beads of Fe_3_O_4_-SH and Fe_3_O_4_@*L-Shikonin* for simultaneous reduction, alkylation, and elution in a one-step process, which was repeated twice. The resulting eluates were pooled, diluted with water, and subjected to enzymatic digestion with trypsin overnight. The digested peptide solution was subsequently desalted using a desalting column, concentrated via centrifugation, reconstituted in loading buffer, introduced into an autosampler, and immobilized on a C18 capture column (3 μm, 120 Å, 100 μm × 20 mm) before elution onto an analytical column (2 μm, 120 Å, 75 μm × 150 mm) for separation. The analytical gradient was established using two distinct mobile phases (mobile phase A: 3% DMSO, 0.1% formic acid, 97% H_2_O and mobile phase B: 3% DMSO, 0.1% formic acid, 97% ACN). The flow rate of the liquid phase was set at 300 nL/min. For mass spectrometry DDA mode analysis, each scan cycle included a full MS scan (R = 70 K, AGC = 3 × 10^6^, max IT = 20 ms, scan range = 350–1800 *m*/*z*) and 15 subsequent MS/MS scans (R = 17.5 K, AGC = 2 × 10^5^, max IT = 100 ms) using a Q Exactive Plus equipped with a high-performance liquid chromatography (HPLC) unit (Thermo Fisher Scientific, Waltham, MA, USA) and a Nanospray Flex Ion-Source (Thermo Fisher Scientific, USA). The HCD collision energy was set to 28, the screening window for the quadrupole was set to 1.6 Da, and the dynamic ion repeat acquisition exclusion time was set to 35 s.

### 2.11. Molecular Docking

The structure of *L-Shikonin* was downloaded from the chemical database, subjected to energy minimization using ChemBio3D 21.0, and underwent hydrogenation and electron addition steps utilizing the Autodock Tools 1.5.6 software. The 3D structure of the target protein was downloaded from the Protein Data Bank (PDB). Dehydration and hydrogenation of the target protein was performed using Autodock; conversion of the receptor and ligand into pdbqt format and molecular docking were performed using Autodock Vina 1.1.2. The interaction between *L-Shikonin* and the target protein were drawn with PyMol 2.0.

### 2.12. Western-Blot

A375 melanoma cells were cultured in a 6-well plate and treated with *L-Shikonin* (2.5, 5.0, and 10.0 μM) for 24 h. Subsequently, total protein was extracted using RIPA buffer containing protease and phosphatase inhibitors at 4 °C. The bicinchoninic acid determination kit (Solarbio, China) measured the protein concentration. RPN1, HNRNPUL1, and CPEB4 were detected using the Western blot protocol. The Western blot signal was quantified using quantity one software.

### 2.13. Statistical Analysis

All data were presented as mean ± standard deviation. In the presence of homogeneity of variance, one-way analysis of variance (ANOVA) was carried out to analyse inter-group comparison. In the presence of variance heterogeneity, the rank test was used for the analysis, and the Student–Newman–Keuls (SNK) method was utilized for the inter-group comparison. *p* < 0.05 was considered to be statistically significant.

## 3. Results

### 3.1. L-Shikonin Inhibited A375 Cell Growth and Induced Apoptosis

**Figure 4:**
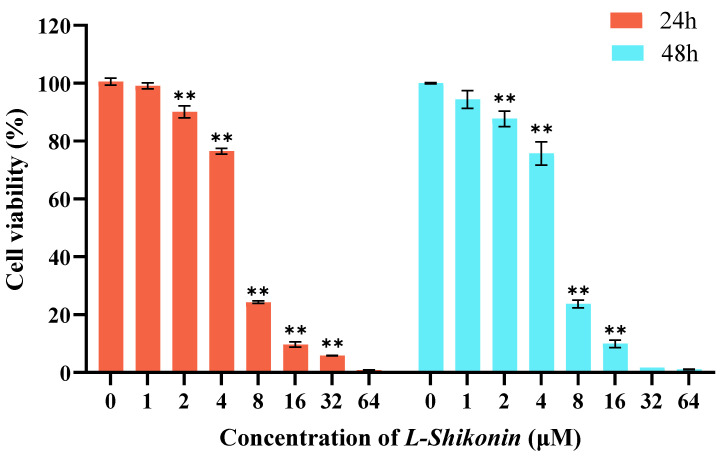
The cell viability of A375 cells treated by *L-Shikonin* was measured by CCK-8 assay. ** *p* < 0.01 vs. Con, n = 3.

**Figure 5:**
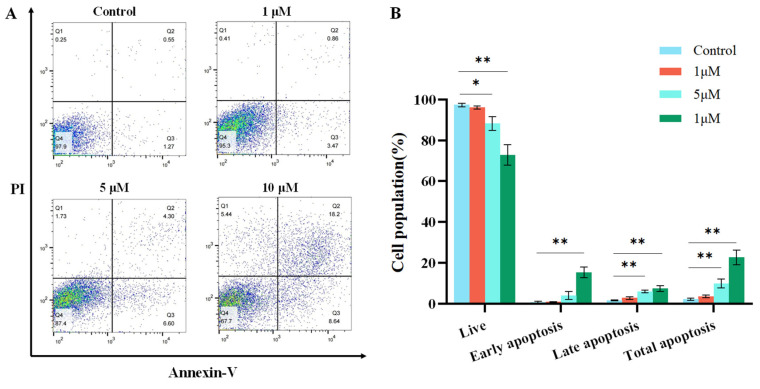
(**A**) Cell apoptosis was analysed via Annexin-V/PI staining. Cells shown in the lower right and upper right represent the percentages of early and late apoptosis, respectively. (**B**) Percentage of cells showing live and apoptosis. * *p* < 0.05, ** *p* < 0.01 vs. Con, n = 3.

**Figure 6:**
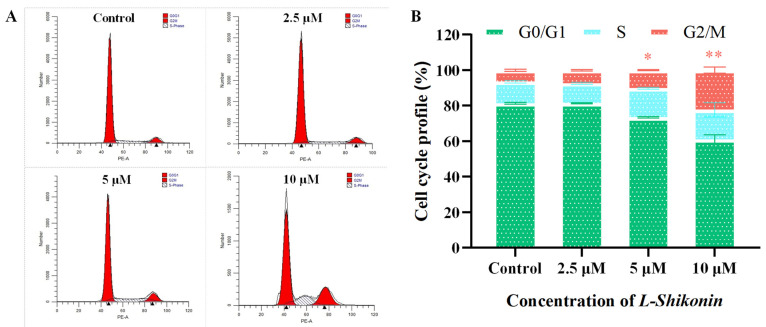
(**A**) A375 cells were treated with *L-Shikonin* (0, 2.5, 5, and 10 μM) for 24 h followed by staining with propidium iodide for flow cytometric analysis. (**B**) The proportion of G0/G1, S, and G2/M phase in the cell cycle. Significant analysis of G2/M phase: * *p* < 0.05, ** *p* < 0.01 vs. Con, n = 3.

The exposure of A375 cells to escalating concentrations of *L-Shikonin* resulted in a dose-dependent inhibition of cell proliferation, as determined by the CCK-8 assay (Figure 4). Significant inhibition of cell viability was observed in the concentration range of 2–64 μM after 24 h of *L-Shikonin* treatment. The IC50 values of *L-Shikonin* treatment at 24 and 48 h were 5.70 ± 0.06 μM and 5.59 ± 0.29 μM, respectively. We also found that the cytotoxicity of *L-Shikonin* on Human Keratinocytes Cells (Hacat) was much lower than that on A375 cells (see Supplementary Figure S1). Moreover, the resistance to apoptosis is a hallmark of cancer cells. The treatment of A375 cells with different *L-Shikonin* concentrations for 24 h resulted in a significant induction of apoptosis, reaching 26.84% at a concentration of 10 μM, as shown in Figure 5. The cell cycle analysis revealed that L-Shikonin treatment led to the arrest of A375 in the G2/M phase (Figure 6). With the increasing concentration of *L-Shikonin* from 2.5 μM and 5 μM to 10 μM, the A375 cell population in the G2/M phase increased to 7.92%, 10.16%, and 24.26% compared with the control group (7.18%). These results demonstrate that *L-Shikonin* has the potential to inhibit the growth and proliferation of A375 cells and induce apoptosis.

### 3.2. Construction of Fe_3_O_4_@L-Shikonin and Its Characterization

**Figure 7:**
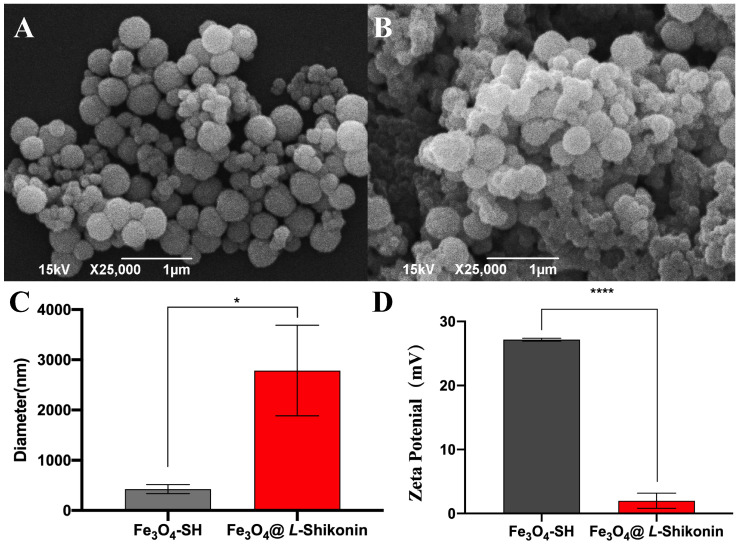
(**A**) SEM images of Fe_3_O_4_-SH magnetic particles, (**B**) SEM images of Fe_3_O_4_@*L-Shikonin* magnetic particles, (**C**) size statistics of Fe_3_O_4_-SH and Fe_3_O_4_@*L-Shikonin* in a Malvern particle sizer, (**D**) potential statistics of Fe_3_O_4_-SH and Fe_3_O_4_@*L-Shikonin*: * *p* < 0.05, **** *p* < 0.0001.

**Figure 8:**
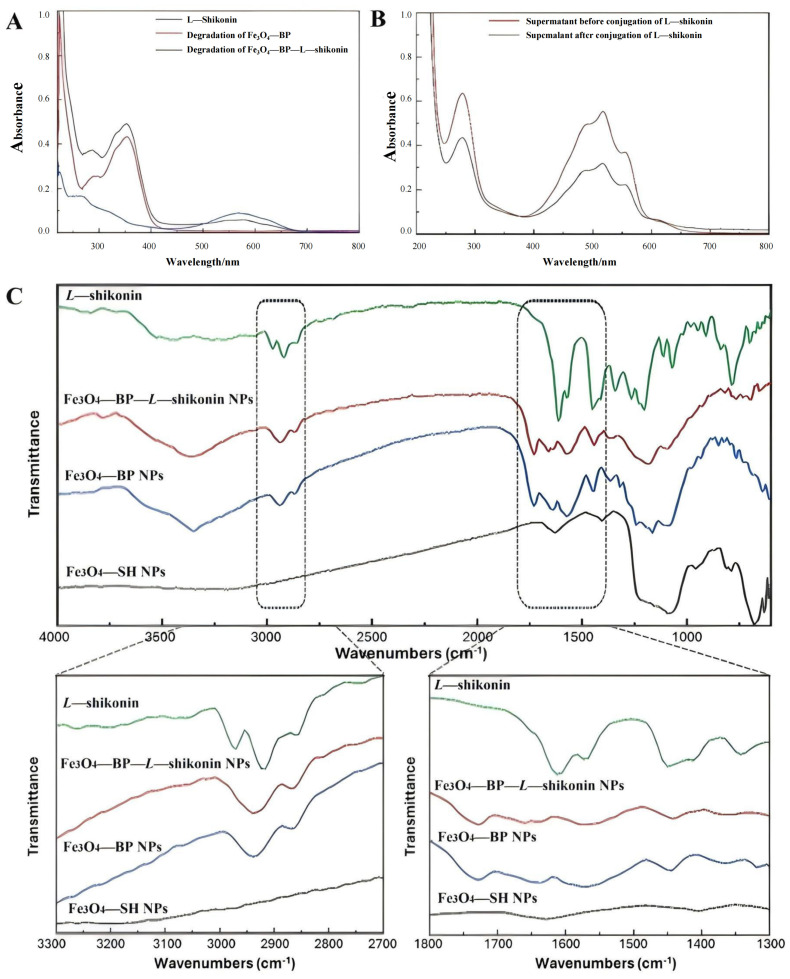
(**A**) Comparison of UV absorption spectra of supernatants before and after coupling *L-Shikonin*. (**B**) UV spectra of base hydrolysis before and after coupling *L-Shikonin*. (**C**) IR spectra before and after coupling *L-Shikonin*.

The Fe_3_O_4_@*L-Shikonin* magnetic particles (Figure 7B) exhibited distinct morphological differences compared to the Fe_3_O_4_-SH magnetic particles (Figure 7A) and displayed a noticeable coupling pattern. Furthermore, an increase in particle size was observed in the Fe_3_O_4_@*L-Shikonin* particles (Figure 7C). The incorporation of *L-Shikonin* led to a reduction in the zeta potential of the magnetic particles (Figure 7D), attributed to the decreased repulsion between particles caused by the polymer and *L-Shikonin* coating on the particle surface. This resulted in a lower potential and larger particle size in the coupled system. FT-IR analysis of Fe_3_O_4_-SH particles in conjunction with *L-Shikonin* on the surface revealed the emergence of new absorption peaks at 1639 cm^−1^ and 1554 cm^−1^ (Figure 8C). After analysis, it was determined that these peaks correspond to the absorption of -C=O and -C-N bonds within the newly formed thiocarbamate resulting from the interaction between -SH and -NCO functional groups. This observation suggests that the polymer particles were synthesized through the reaction of these functional groups. Additionally, asymmetric and symmetric stretching vibrations of methyl and methylene were observed at 2936 cm^−1^ and 2863 cm^−1^, respectively. New peaks were generated in the spectrum after coupling *L-Shikonin*, including a C=C bonding peak at 1442 cm^−1^ and C-H bending vibrations at 1353 and 1080 cm^−1^. The intensity of the ketone carbonyl peak at 1643 cm^−1^ in the BP group was significantly reduced after coupling *L-Shikonin*. *L-Shikonin* and *L-Shikonin* coupled with Fe_3_O_4_-BP NPs had similar spectral peaks, indicating successful coupling.

To further analyse the drug molecules coupled on the surface of magnetic particles, the polymer degradation method was utilized. The grafted polymer and the coupled *L-Shikonin* were removed from the surface of magnetic particles by alkali hydrolysis, and then the *L-Shikonin* bonded on the surface of magnetic particles was analysed by UV-Vis spectroscopy. The spectrum shows that the BP group in the graft polymer has obvious absorption peaks near 280–300 nm and 350 nm, and *L-Shikonin* has obvious absorption peaks at 310 nm and 550–650 nm. This indicates that the alkali hydrolysate contains both polymer and *L-Shikonin*, confirming the coupling of *L-Shikonin* on the surface of the magnetic particles (Figure 8B).

The supernatant analysis method was utilized to examine the UV-Vis spectra of dispersions containing Fe_3_O_4_-BP particles and drug molecules before and after illumination. Additionally, the variation in *L-Shikonin* content in the supernatant pre- and post-illumination was assessed, confirming that the drug molecules bound to the magnetic particles’ surface are present at a concentration of at least 87.13 mM (Figure 8A). It is important to note that the presence of other substances in the supernatant analysis method may introduce potential interference at 518 nm, potentially leading to elevated detection results. In the polymer degradation assay, the bonded *L-Shikonin* may not have been completely dissociated, resulting in a low level of detection.

### 3.3. Proteomic Analysis of L-Shikonin Hook Fishing Based on High-Resolution Mass Spectrometry

**Figure 9:**
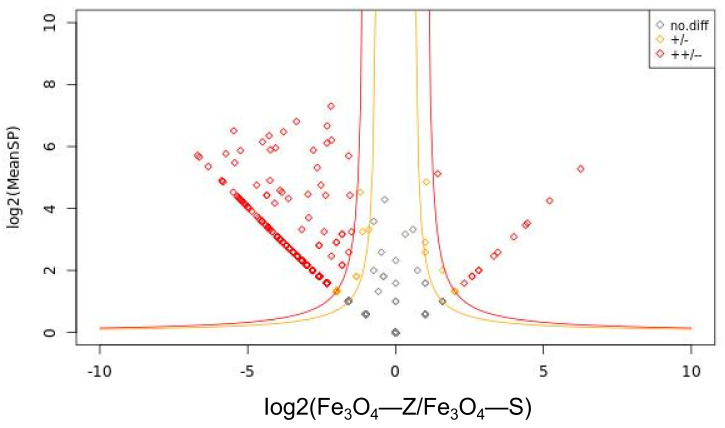
Differential protein screening. The yellow and red lines are the screening boundaries for differential proteins.

A total of 780 proteins were identified on Fe_3_O_4_-SH magnetic beads, while 145 proteins were identified on Fe_3_O_4_@*L-Shikonin* magnetic beads. A scatter plot was generated by computing the ratio of the number of spectra (ratio) and the mean number of spectra (MeanSP) for each protein across various sample groups (Figure 9). The log2 ratio was utilized as the horizontal coordinate, and log2 MeanSP was utilized as the vertical coordinate. Protein-screening boundary lines were set according to the scatter distribution of individual proteins, and the statistical significance of differences was indicated. Different proteins were screened according to log2 ratio and log2 MeanSP, and the significance of differences was marked. The statistical quantities of different proteins are shown in Table 1. Among the differential proteins identified in the Fe_3_O_4_@*L-Shikonin* and Fe_3_O_4_-SH, 7 proteins exhibited up-regulation, 13 proteins showed significant up-regulation, 69 proteins displayed down-regulation, 267 proteins demonstrated significant down-regulation, and 467 proteins did not show significant differences. The primary focus of this study was on the 20 up-regulated proteins. A total of 44 unique proteins were identified on Fe_3_O_4_@*L-Shikonin* magnetic beads, with 12 of these proteins overlapping with the 20 up-regulated proteins. Consequently, these 52 proteins were selected for further investigation. The molecular docking of *L-Shikonin* acid with each of the 52 proteins was subsequently validated (Table 2).

**Figure 10:**
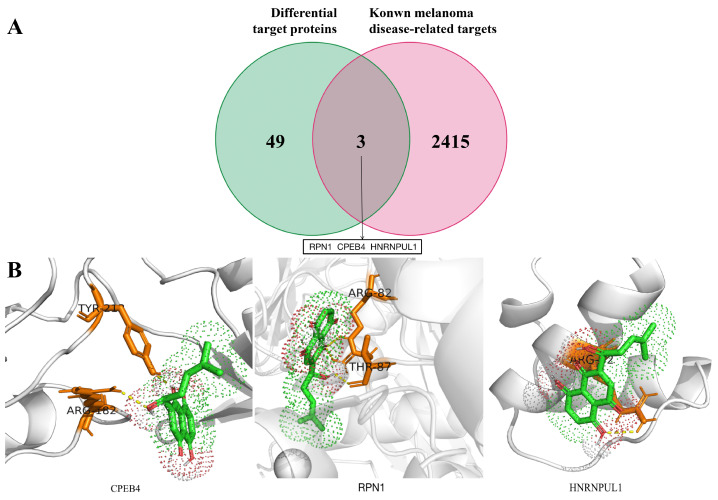
(**A**) Venn diagram of Fe3O4@*L-Shikonin* hooked differential protein and melanoma-related targets. (**B**) Molecular docking diagram of *L-Shikonin* with CPEB4, RPN1, and HNRNPUL1.

The results of the docking analysis (Table 1) revealed that 20 out of the 52 proteins lacked a crystal structure or could not generate a docking simulation due to steric clashes. The ligand molecule successfully docked with 28 proteins with a minimum binding energy below −5 kJ∙mol^−1^, indicating favourable binding. Fe_3_O_4_@*L-Shikonin* interacted with 52 differential target proteins, which were further intersected with known melanoma-related targets (Figure 10A). Three intersected targets, namely RPN1, CPEB4, and HNRNPUL1, exhibited docking energies of −7.9, −6.4, and −5.4 kJ∙mol^−1^, respectively, all falling within the subset of 28 proteins with promising molecular docking results. However, these findings need to be verified in further experiments. Among the 28 proteins, *L-Shikonin* potentially exerts its anti-melanoma effects by modulating invasion, migration, apoptosis, and melanocyte proliferation. Of these, we focused our analysis on three targets known to be associated with melanoma, CPEB4, RPN1, and HNRNPUL1.

CPEB4 is a protein that binds specifically to RNA. Studies have shown that CPEB protein is abnormally expressed in various tumour cells and tissues, partially overlaps and affects cancer cells with other genes or pathways (e.g., p53, tPA, Bcl-xL/Bax, MAPK/ERK pathway, etc.), affects the proliferation, migration, and invasive ability of cancer cells, and participates in the regulation of the tumour epithelial–mesenchymal transition (EMT) process, and many studies have suggested that CPEB4 may be closely related to tumorigenesis and development [36]. Tumours of different tissue origins may have different expressions of CPEB4 due to tissue specificity. For example, Chen et al. [37] found that the mRNA and protein expression of CPEB4 was upregulated in astrocytic tumour tissues, and the results of Xu et al. [38] showed that CPEB4 was lowly expressed in pancreatic ductal adenocarcinoma, intraductal papillary mucinous adenoma, and intraductal papillary mucinous carcinoma. Its molecular docking with *L-Shikonin* (Figure 10B) showed good docking results with a binding energy of −6.4 kJ∙mol^−1^. Researchers from the Spanish National Cancer Research Centre have identified the CPEB4 protein as a “pioneer” in melanoma development, influencing the process of melanoma formation. They have found that CPEB4 can induce polyadenylation of the mRNAs of MITF and RAB27A and thus regulate their expression, thereby controlling the proliferation and metastasis of melanoma [39].

RPN1 is a subunit of the oligosaccharyltransferase (OST) complex that catalyses the initial transfer of the eukaryotic identified glycan (Glc(3)Man(9)GlcNAc(2)) from the lipid carrier polyglycol-pyrophosphate to the Ser/Thr common motif in the asparagine residue-nascent polypeptide chain within Asn-X, the first step in protein N-glycosylation; N-glycosylation occurs by co-translation, and the complex binds to the Sec61 complex at the channel forming a translocator complex, which mediates the translocation of the protein through the endoplasmic reticulum (ER). Translation occurs, and the complex binds to the Sec61 complex at the channel to form a translocator complex, which mediates protein translocation through the ER. Melanin is the cause of almost all visible skin, hair, and eye pigmentation in humans and is synthesized, deposited, and distributed in subcellular organelles called melanosomes. In humans, melanin in the skin, hair, and eyes protects the body from environmental challenges such as solar UV exposure, toxic free radicals, and heavy metals. Variations in the chemical composition, structure, and distribution of melanosomes result in different skin, hair, and eye colour variations in a population. Pigmentation and dysfunction of melanosome biosynthesis are associated with a variety of genetically inherited diseases and pigmentary disorders, including oculocutaneous albinism and Hermansky–Pudlak syndrome. Melanosome-specific proteins also provide important markers for malignant melanoma. It has been proven in studies [40,41] that RPN1 is associated with melanosomes and that its molecular docking with *L-Shikonin* (Figure 10B) has a binding energy of −7.9 kJ∙mol^−1^ and gives good docking results.

The heterogeneous nuclear ribonucleoprotein (hnRNP) superfamily, consisting of over 20 identified members from A1 to U, is a widely distributed group of nuclear proteins found in plants and animals. Extensive research has demonstrated the essential role of hnRNPs in mammalian development, specifically in processes such as pre-RNA splicing, mRNA transport, localization, and translation. HNRNPUL1, a recently discovered member of the hnRNP family, has been shown to interact with mRNAs, influencing RNA transport and processing and promoting DNA damage by activating various signalling pathways [42]. The association of HNRNPUL1 with tumours has not yet been investigated; however, it has been linked to tyrosine kinases, [43] which may subsequently govern the growth and spread of melanoma cells. The molecular docking analysis of HNRNPUL1 with *L-Shikonin* (Figure 10B) exhibited a favourable binding energy of −5.4 kJ∙mol^−1^.

Studies on the effects of *L-Shikonin* on the above proteins and melanoma are limited and need to be validated by further experiments. In this study, molecular docking analysis was performed to investigate the binding of *L-Shikonin* to multiple cellular targets that are highly correlated with various functions. The analysis also revealed that *L-Shikonin* can form strong interactions with these targets through hydrogen bonds.

### 3.4. Effect of L-Shikonin on the Relative Expression of CPEB4, HNRNPUL1, and RPN1 in A375 Cells

**Figure 11:**
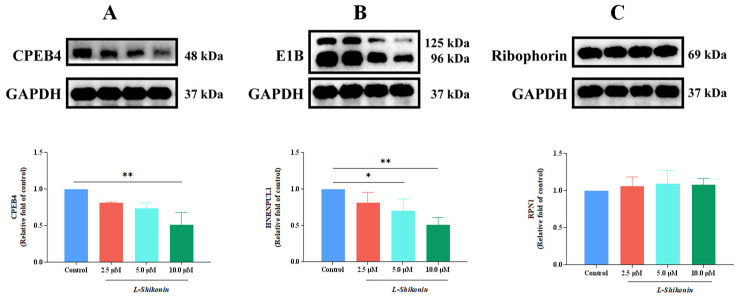
(**A**) Effect of *L-Shikonin* on the expression of protein CPEB4. (**B**) Effect of *L-Shikonin* on the expression of protein HNRNPUL1. (**C**) Effect of *L-Shikonin* on the expression of protein RPN1. * *p* < 0.05, ** *p* < 0.01 vs. Con, n = 3.

Following treatment with *L-Shikonin*, the relative expression of CPEB4 exhibited a decrease corresponding to the dosage of *L-Shikonin* administered. In comparison to the control group, a statistically significant reduction in the relative expression of CPEB4 was observed in the high-dose group (*p* < 0.01) (Figure 11A). Similarly, the relative expression of HNRNPUL1 decreased in response to increasing *L-Shikonin* dosage, with statistically significant decreases observed in the medium-dose group and high-dose group compared to the control group (*p* < 0.05) (Figure 11B). Conversely, *L-Shikonin* did not result in a significant change in the relative expression of RPN1 (Figure 11C).

## 4. Discussion

In this study, magnetic microspheres of *L-Shikonin* were synthesized and used to capture the target protein group associated with *L-Shikonin*. Analysis of the target proteins revealed the identification of 145 proteins bound to the *L-Shikonin*-bonded magnetic solid-phase microspheres. A total of 52 proteins exhibiting differences were selected as potential candidate targets from the initial pool of 145 targets. Molecular docking studies were then conducted between *L-Shikonin* and the 52 proteins, identifying 28 proteins with minimum binding energies below −5 kJ∙mol^−1^. Simultaneously, three of these proteins intersected with established melanoma-related targets, namely RPN1, CPEB4, and HNRNPUL1. CPEB4 plays a role in modulating the proliferation and metastasis of melanoma cells, and HNRNPUL1 is implicated in regulating tyrosine kinases linked to aberrant tyrosine metabolism in melanoma. Ultimately, the anti-melanoma efficacy is achieved through the modulation of multiple targets.

However, this study only identified three primary target proteins and corresponding pathways of *L-Shikonin* in melanoma through bioinformatics analysis, molecular docking, and known melanoma-related targets and does not represent all the target proteins and pathways of *L-Shikonin* against melanoma. This limited analysis does not encompass all potential target proteins and pathways of *L-Shikonin* against melanoma. Other proteins that have not been analysed may also serve as target proteins, and further verification through further experiments at the cellular and protein levels is warranted. Additionally, the spatial conformation of *L-Shikonin* may be altered when attached to magnetic beads, potentially resulting in variances in the proteins bound by Fe_3_O_4_@*L-Shikonin* compared to those directly influenced by *L-Shikonin* within cellular environments. While the influence of Fe_3_O_4_@*L-Shikonin* on the conformation of *L-Shikonin* may be insignificant, unsuccessful bonding could arise due to multiple factors, warranting additional experimentation for elucidation.

Both this study and traditional target discovery methods necessitate structural modification of small molecules to identify target proteins. However, such modifications can potentially result in false-positive outcomes due to non-specific binding. The emergence of proteomics, mass spectrometry, and bioinformatics has paved the way for the utilization of label-free small-molecule probes as a novel approach for natural product target discovery. The notable features of this approach include its ability to accommodate complex chemical components of Chinese medicine without necessitating structural modifications. Additionally, the method is grounded in proteomic target screening, thereby aiding in the identification of novel targets and pathways of action for Chinese medicine. Current label-free small-molecule probe methods encompass drug affinity-related target stability techniques, thermal proteomic analysis techniques, and protein oxidation rate stability analysis techniques. The label-free small-molecule probe technology reduces the barrier to target the discovery of natural compounds by eliminating the necessity for modification and derivatization of ligand compounds. This method offers a rapid, accurate, and straightforward approach to identifying target proteins of natural product components, thereby facilitating the advancement of natural product research towards a stage of rapid development centred around target identification.

## 5. Conclusions

This study elucidated that *L-Shikonin* may regulate melanoma-specific markers, melanosomes, tyrosine kinases related to abnormal tyrosine metabolism, and melanoma through multiple targets such as CPEB4 and HNRNPUL1. Proliferation and metastasis work together to exert an anti-melanoma mechanism, which provides a new idea for the follow-up study of the molecular pharmacological mechanism of the complex system of total naphthoquinones in *Arnebia euchroma* (Royle) Johns.

## Figures and Tables

**Table 1:** Differential protein statistics.

diff.sig	Fe_3_O_4_-Z/Fe_3_O_4_-S
+	7
++	13
-	69
--	267
no.diff	467

**Table 2:** Molecular docking binding energy of *L-Shikonin* and its targets.

No.	PDB ID/UniProt ID	Protein Name	Binding Energy (kJ∙mol^−1^)	No.	PDB ID/UniProt ID	Protein Name	Binding Energy (kJ∙mol^−1^)
1	4PJ3	AQR	−8.8	27	5O3J	TIA1	−5.7
2	6O5F	DDX3X	−8.7	28	1ZRJ	HNRNPUL1	−5.4
3	6F1W	CSNK1D	−8.5	29	O15027	SEC16A	−4.2
4	6S7O	DDOST	−8.1	30	Q13310	PABPC4	−3.7
5	6S7O	RPN1	−7.9	31	6WTT	HNRNPDL	−3.6
6	5W6V	TNRC6A	−7.9	32	6BXX	HNRNPA1	34.6
7	6UDP	IMPDH2	−7.7	33	/	SRSF10	/
8	6PU1	SEC24C	−7.4	34	/	SLC25A5	/
9	5O9Z	PRPF4	−7.4	35	/	U2AF1L5	/
10	5VNO	SEC24A	−7.2	36	/	RPS4Y1	/
11	5VM9	AGO3	−7.1	37	/	AGL	/
12	7D3X	APOBEC3A	−7	38	/	RPL21	/
13	3KUT	PAIP2	−6.9	39	/	MRPS22	/
14	2DH7	TIAL1	−6.8	40	/	MRPS5	/
15	6WMG	SUN2	−6.8	41	/	MRPS34	/
16	6DHS	HNRNPH1	−6.6	42	/	HNRNPUL2	/
17	1ZKC	PPIL2	−6.5	43	/	ZCCHC8	/
18	7C36	RBMS1	−6.5	44	/	PTCD3	/
19	2MKJ	CPEB4	−6.4	45	/	WDCP	/
20	3S7R	HNRNPAB	−6.4	46	/	KCMF1	/
21	2DNP	RBM14	−6.3	47	/	AGO2	/
22	5IM0	HNRNPD	−6.3	48	/	AKAP8L	/
23	2KG1	HNRNPF	−6.3	49	/	TNRC6B	/
24	2DNQ	RBM4	−6	50	/	MRPS18B	/
25	1QOI	PPIH	−5.9	51	/	RPL6	/
26	5HO4	HNRNPA2B1	−5.9	52	/	ILF2	/

## Data Availability

The raw data supporting the conclusions of this article will be made available by the authors upon request.

## Supplementary Materials

The following supporting information can be downloaded at: https://www.mdpi.com/article/10.3390/pharmaceutics16121543/s1, Figure S1. Cytotoxicity of *L-Shikonin* on Hacat cells. Cells were treated with *L-Shikonin* at 0–64 μM for 24 h, and cell viability was determined by cck-8 assay and analyzed by Gaphpad prism software (10.1.2).
