# Supplementary material for: Global Identification of Anti-Melanoma Cellular Targets by Photochemically Induced Coupling of L-Shikonin Reactions on the Surface of Magnetic Particles
Source: Pharmaceutics. 2024 Dec 2;16(12):1543. doi: 10.3390/pharmaceutics16121543 (PMC11728473; doi:10.3390/pharmaceutics16121543)
Supplement: Supplementary file 1 [file pharmaceutics-16-01543-s001.zip › pharmaceutics-3307703-supplementary.pdf]

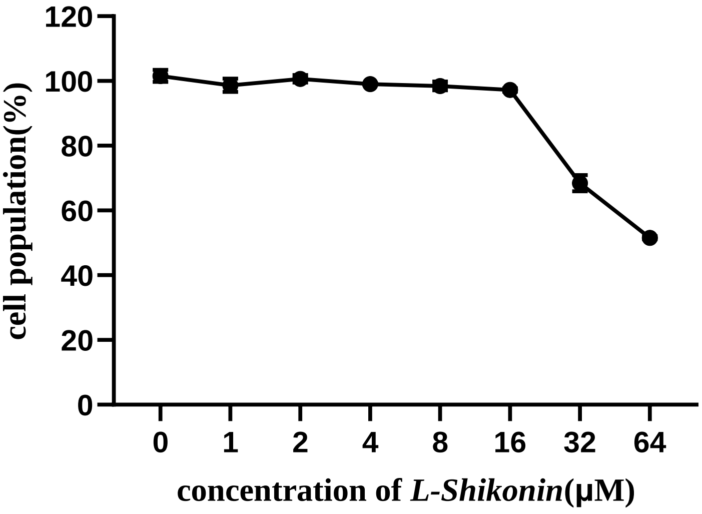

Figure S1 Cytotoxicity of *L-Shikonin* on Hacat cells. Cells were treated with *L-Shikonin* at 0-64μM for 24h, and cell viability was determined by cck-8 assay and analyzed by Gaphpad prism software(10.1.2).
